# Salivary gland tumors: A 13-year clinicopathologic retrospective study in a Brazilian northeast population

**DOI:** 10.4317/jced.59738

**Published:** 2023-02-01

**Authors:** John-Lennon-Silva Cunha, Vitória-Ribeiro-Almico Fraga, Wliana-Pontes de Lima, Allany-de Oliveira Andrade, Manuel-Antonio Gordón-Núñez, Cassiano-Francisco-Weege Nonaka, Pollianna-Muniz Alves, Ricardo-Luiz-Cavalcanti-de Albuquerque Júnior

**Affiliations:** 1 DDS, MSc, Professor. Department of Dentistry, State University of Paraíba (UEPB), Campina Grande, PB, Brazil; 2 DDS,MSc student Laboratory of Morphology and Experimental Pathology, Institute of Technology and Research, Tiradentes University (UNIT), Aracaju, SE, Brazil; 3 DDS, MSc, PhD student. Postgraduate Program in Dentistry, Department of Dentistry, State University of Paraíba (UEPB), Campina Grande, PB, Brazil; 4 DDS, PhD, Professor. Department of Dentistry, State University of Paraíba (UEPB), Araruna, PB, Brazil; 5 DDS, PhD, Professor. Department of Dentistry, State University of Paraíba (UEPB), Campina Grande, PB, Brazil; 6 DDS, PhD, Professor. Laboratory of Morphology and Experimental Pathology, Institute of Technology and Research, Tiradentes University (UNIT), Aracaju, SE, Brazil

## Abstract

**Background:**

The present study aimed to evaluate the clinicopathologic features of salivary gland tumors (SGTs) in a Brazillian northeast population.

**Material and Methods:**

A retrospective descriptive cross-sectional study was performed (1995-2009). All cases of SGTs diagnosed in a private surgical pathology service in Brazil were reviewed, and clinicopathological data were collected.

**Results:**

A total of 23.258 histopathological records of biopsies were analyzed, and 174 cases were diagnosed as SGTs (0.7%). Of these, 117 (67.2%) were classified as benign, and 57 (32.8%) were malignant. The series comprised 89 females (51.1%) and 85 males (48.9%), with a mean age of 50.2 years (range: 3-96 years) and a roughly equal female-to-male ratio (1:1). Most tumors occurred in the parotid gland (n = 82, 47.1%), followed by the palate (n = 45, 25.9%), submandibular gland (n = 15, 8.6%). Pleomorphic adenoma (n = 83; 70.9%) and mucoepidermoid carcinoma (n = 19, 33.3%) were the most frequent benign and malignant tumors, respectively. After reevaluation of morphology and immunohistochemical analysis, seven tumors (4.0%) were reclassified following the current WHO Classification of the Head and Neck Tumors.

**Conclusions:**

The general features of SGT from the studied Brazilian population were similar to previously published reports in other countries. However, SGTs do not show any sex predilection. Although careful morphological analysis is the key to the correct diagnosis of these tumors, immunohistochemical analysis is essential to establish an accurate diagnosis in the face of challenging cases.

** Key words:**Salivary gland tumors, epidemiology, head and neck pathology.

## Introduction

A variety of tumors can develop in the salivary glands (1,2). Currently, the World Health Organization (WHO) has published some changes in the classification of SGTs, recognizing several new entities such as sclerosing polycystic adenoma, keratocystoma, intercalated duct adenoma, and striated duct adenoma among the benign neoplasms; and microsecretory adenocarcinoma and sclerosing microcystic adenocarcinoma as new malignant entities (2). Despite of a large number of histological subtypes, these tumors account for only approximately 3 to 6% of all tumors in the head and neck region, with an estimated global incidence of 0.4 to 13.5 per 100,000 people annually (2-5). Considering their wide histological variety and different biological behaviors, knowing their clinical and pathological characteristics and the incidence is essential to establishing the proper management and prognosis (1,3,4).

Brazil is the largest South American country in population, with an estimated population of approximately 211.8 million people, according to the latest census Figures and projections from Trading Economics (2020). However, there is a dearth of literature on the frequency and distribution of SGTs in the Northeast Region of Brazil, particularly in the state of Sergipe. To the best of our knowledge, this is the second study on salivary gland tumors in the state of Sergipe, Northeast Brazil (3).

Although several epidemiological studies have been carried out in different parts of the world and provide valuable knowledge (1,3-15), the incidence of salivary gland tumors varies among other geographic regions, with discrepancies between the clinicopathological aspects, especially regarding the anatomical location and histological subtypes (1,3,4,8). Therefore, local records are a helpful strategy for analyzing the distribution and particular characteristics of SGTs in a specific population (6), contributing to the establishment of an early diagnosis, adequate treatment, and cancer prevention.

Thus, the present study aimed to describe the clinicopathologic features of SGTs diagnosed in a Brazillian private surgical pathology service and compare the findings with epidemiological data from different geographic locations.

## Material and Methods

-Ethical aspects

 The study was approved by the Ethical Committee of Tiradentes University (Protocol nº 87722518.3.0000.5371).

-Study design and sample

In this study, the archives of Nestor *Pi*va Memorial in Aracaju City (Sergipe State, Brazil) were retrospectively reviewed. During a 13-year period, between January 1995 and December 2009, all cases of SGTs were retrieved from this archive. Five-micrometer hematoxylin and eosin-stained sections were obtained from each case, and all oral pathologists included in the study re-evaluated the histological features of the tumors. The tumors were reclassified into benign and malignant tumors in accordance with the current WHO Classification of the Head and Neck Tumors (2022) (2). Disagreements between the examiners were solved upon discussion and reaching a consensus. Patients’ age, sex, anatomical location, and histopathological diagnosis were obtained from clinical records and evaluated. Immunohistochemical and histochemical analyses were performed when routine staining (hematoxylin and eosin) was not sufficient to establish the final diagnosis.

-Analysis

Descriptive and quantitative data analysis was performed using the Statistical Package for the Social Sciences for Windows 20.0 (SPSS, Inc., Chicago, IL, USA). Continuous variables were expressed as mean, median, and standard deviation values. Categorical variables were expressed as the absolute number of cases and percentage values. Person’s chi-square test and Fisher’s exact test evaluated the association between biological behavior (malignant vs. benign tumors) and clinical and demographic characteristics, adopting a *p-value* of ≤ 0.05 and a 95% confidence interval.

## Results

A total of 23.258 histopathological records of biopsies were analyzed between 1995-2009, of which 174 were diagnosed as SGTs (0.7%). Of the total of 174 cases of salivary gland tumors, 117 (67.2%) were benign, and 57 (32.8%) were malignant tumors with a benign to malignant ratio of 2.1:1, distributed among seven benign and ten malignant histologic subtypes (Table 1).

**Table 1:**
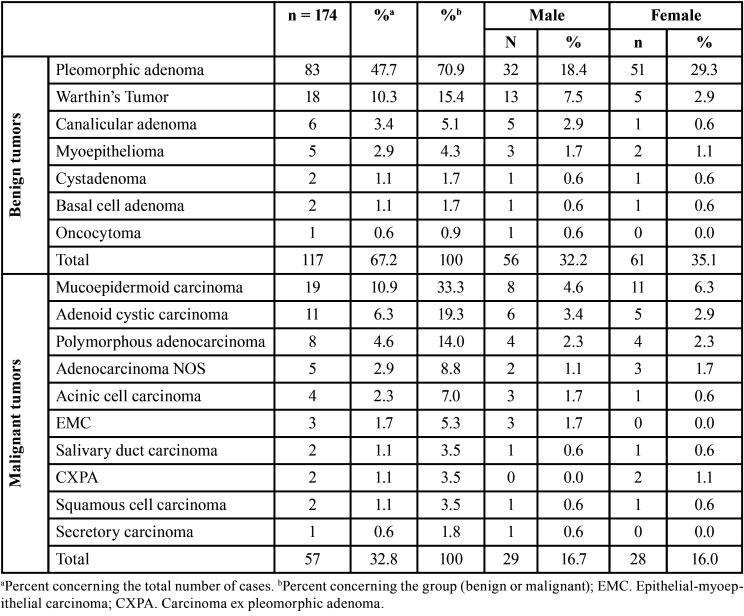
Histologic and sex distribution of 174 salivary gland tumors.

Most tumors occurred in the patients in the fourth and seventh decades of life, with a mean age of 50.2 years (range 03-96 years). A similar female-to-male ratio (1:1) was observed for benign and malignant tumors (Table 1). The distribution of each salivary gland tumor according to patients’ age is shown in Table 2. Regarding the site, most of the tumors occurred in the major salivary glands (n = 97, 55.7%), while 41.4% (n = 72) affected the minor salivary glands. The parotid gland was the most commonly affected site (n = 82, 47.1%), followed by the palate (n = 45, 25.9%), submandibular gland (n = 15, 8.6%), and buccal mucosa (n = 13, 7.5%). There were five cases with unspecified anatomic locations (2.9%). No tumor affected the sublingual gland. Both benign and malignant tumors predominated in the parotid gland (Table 3).

**Table 2:**
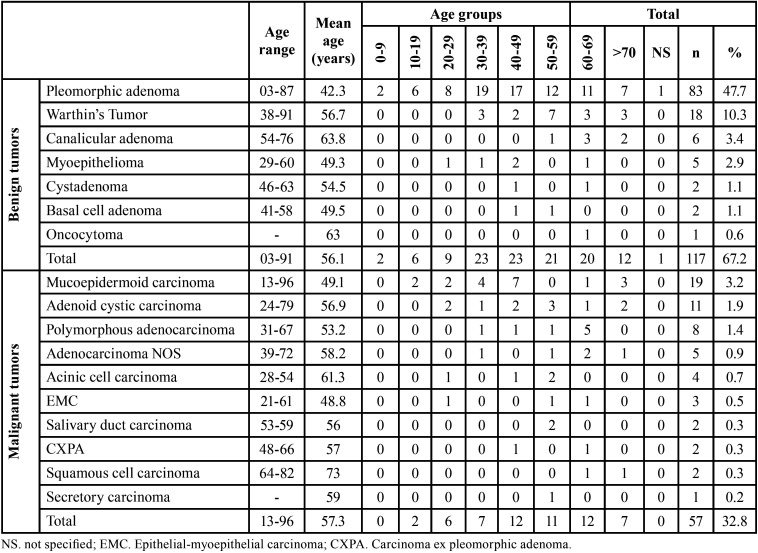
Age group distribution (decade of life) of 174 salivary gland tumors.

**Table 3:**
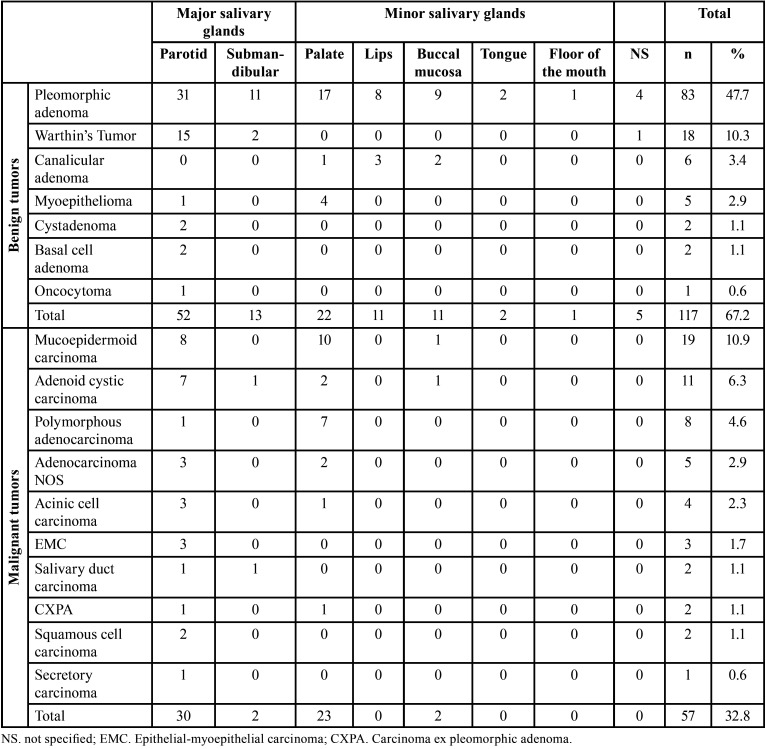
Distribution of the salivary gland tumors according to the location (major and minor salivary glands).

**Figure 1:**
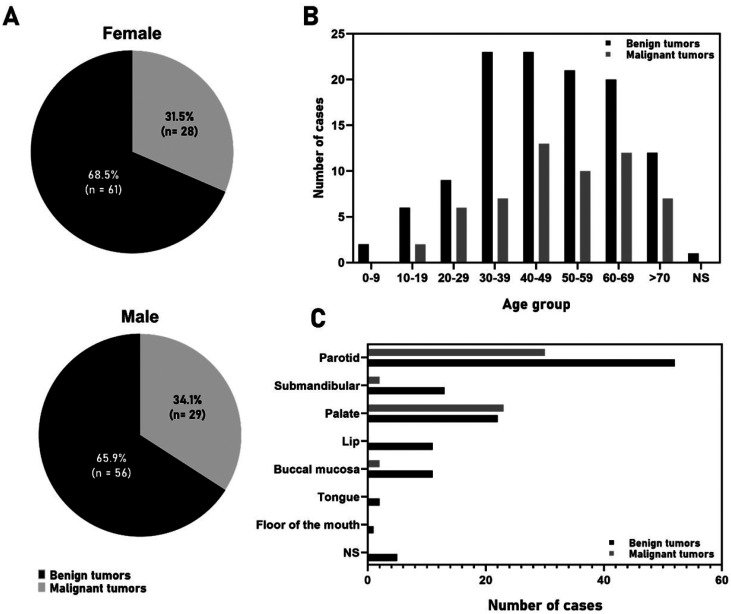
Distribution of 174 salivary gland tumors according to (A) sex, (B) age group (decade of life), and (C) primary site of involvement. NS, not specified.

Among the benign salivary gland tumors, pleomorphic adenoma (PA) was most frequent (n = 83; 70.9%), followed by Warthin’s tumor (n = 18, 15.4%), and canalicular adenoma (n = 6, 5.1%) (Table 1). These tumors were diagnosed mainly between the fourth and seventh decades of life (Fig. 1); however, the age ranged from 03 to 91 years, with a mean age of 56.1 years (SD ± 18.7) (Table 2). Most cases occurred in the parotid gland (n = 52, 44.4%) and female patients (n = 61; 52.1%), with a female:male ratio of 1.1:1 (61 female and 56 male).

Regarding the malignancies, mucoepidermoid carcinoma (MEC) was the most frequent malignant tumor (n = 19, 33.3%), followed by adenoid cystic carcinoma (ACC) (n = 11, 19.3%), and polymorphous adenocarcinoma (n = 8, 14.0%) (Table 1). The patient’s ages ranged from 13 to 96 years, with a mean age of 57.3 years (SD ± 22.1) (Table 2). Malignant tumors do not show sex predilection (female:male ratio of 1:1). Most cases occurred in the parotid gland (n = 30, 52.6%), followed by minor salivary glands of the palate (n = 23, 40.4%).

In twenty-one (12.1%), histochemical analysis was carried out to aid in the diagnosis. Different histochemical stains, such as Periodic Acid-Schiff (PAS), mucicarmine, and alcian blue staining, were used to determine the nature of the mucinous material and aid in the diagnosis. Immunohistochemical reactions (IHC) were used in 9 cases (5.2%). In 5 cases, IHC was used to determine the proliferative index; in only 4 cases, it aimed to identify cells and structures to facilitate the diagnosis.

After reevaluation of morphology and immunohistochemical studies, seven tumors (4.0%) were reclassified following the current WHO Classification of the Head and Neck Tumors (2). Among the benign SGTs, two cases previously diagnosed as PAs were reclassified as carcinoma ex-pleomorphic adenomas (CXPA). Regarding the malignancies, five adenocarcinoma NOS (AcNOS) were reclassified, two cases as polymorphous adenocarcinomas, two cases as mucoepidermoid carcinomas, and one case as secretory carcinoma. One polymorphous adenocarcinoma was compatible with cribriform adenocarcinoma of minor salivary glands (CAMSG), a variant of polymorphous adenocarcinoma. However, the case consistent with CAMSG was maintained as polymorphous adenocarcinoma based on the current WHO Classification of Head and Neck Tumors (2).

There is no significant association between the biologic behavioral (malignant versus benign tumors) and clinical and demographic characteristics (*P* > 0.05) (Table 4).

**Table 4:**
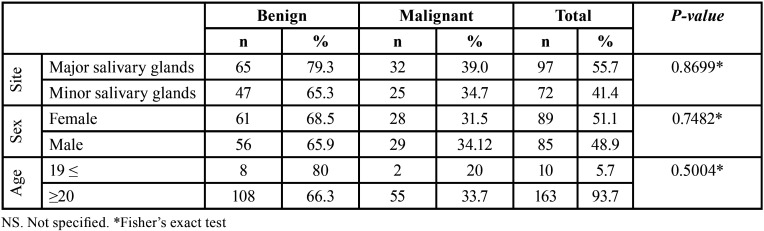
The relationship between biologic behavior and clinicopathological characteristics of salivary gland tumors.

## Discussion

In the last few decades, many studies have demonstrated the frequency of SGTs in all continents (1,3-14,16-19). However, variations in frequency were observed depending on referral sources and type of diagnostic services (private, public, hospital, etc.) (1). In the present investigation, the sample represented about 0.7% of the total lesions diagnosed in the referred service. Studies in other pathology services reveal that SGTs account for about 0.08% (12) to 19.6% (13) of all diagnosed lesions, data similar to our results.

According to the literature, female patients are slightly more affected by SGTs than male patients (2). However, some variations can be found when analyzing specific tumor subtypes (1,3,4). A similar male-to-female ratio (1:1) was observed for benign and malignant tumors in the present study. Although uncommon, Vasconcelos *et al*. (6), Lukšić *et al*. (30), and Tian *et al*. (5) reported similar findings. On the other hand, some reports show that men are mainly affected by malignant SGTs (11), including studies from Brazil (3,4).

This study showed a high predominance of benign (67.2%) over malignant tumors (32.8%), similar to most previous studies on SGTs (1,3-15). However, some studies conducted on the African (16,17) and Asian continents (13,18) have shown a higher incidence of malignant tumors. The possible reason for the high number of malignancies could be that since most African reports are from tertiary referral centers (16,17), they receive a disproportionate number of malignant lesions from outlying centers. Thus, the study’s place might explain the divergence of these results.

SGTs are found across all age groups (1,3,4,8,9). In this study, the age range of patients varied from 3 to 96 years, with a mean age of 50.2 years. In addition, individuals in the fourth to seventh decades made up 70% of total patients, similar to previous studies (3,4,16). The mean age of patients with benign tumors was 56.1 years, similar to other studies (3,4,6,16). Malignant tumors showed a mean slightly larger than benign tumors (57.2 years) but were not statistically significant (*p* > 0.05). The difference between the mean age of malignant and benign tumors has been reported to vary from 3 to 10 years (4,16). However, some previous reports, including the present study, have found an almost equal average age between malignant and benign tumors (9,11).

PA was the most common tumor in this study, accounting for 70.9% of all benign tumors, followed by Warthin’s tumor (15.4%) and canalicular adenoma (5.1%). Previous studies clearly show that PA is the most common benign neoplasm in major and minor salivary glands (1,3-15). However, in contrast to our results, some studies have shown myoepithelioma (13,17) or basal cell adenoma (4) as the second most common benign tumor. Overall, the most frequent benign tumors are PA, Warthin’s Tumor, basal cell adenoma, and myoepithelioma (3).

Regarding the malignant tumors, MEC was the most frequent tumor accounting for 33.3% of the cases, followed by ACC (n = 11, 19.3%) and polymorphous adenocarcinoma (n = 8, 14.0%). Several studies have shown MEC as the most common malignant salivary gland tumor (3,4,7-9,11,14); however, other studies have reported ACC as the most prevalent malignant tumor (5,6,10). Few previous reports showed polymorphous adenocarcinomas among the three most common malignant salivary gland tumors (1,12). Also, in our study, some other malignant tumors were very rare, such as secretory carcinoma (n = 1, 1.8%), squamous cell carcinoma (n = 2, 3.5%), salivary duct carcinoma (n = 2, 3.5%), and carcinoma ex pleomorphic adenoma (n = 2, 3.5%) in accordance with previous studies (3,4,8).

The difference in the frequency of these tumors varies significantly in the literature (1,3). It may be explained due to complex definition, a great diversity of morphologic features, different classifications, low prevalence, and time of experience and familiarity of pathologists with these lesions (1,3,4). In addition, several studies have shown considerable inter-observer variations in morphology assessment between pathologists (19). In the present study, the morphologic diagnosis of all tumors was re-evaluated according to the current WHO classification (2017) (2). Seven cases (4.0%) were reclassified based on morphological characteristics and immunohistochemical studies. Of these, two cases of PAs were reclassified as CXPAs.

Histologically, most CXPAs clearly show the transition of benign PA into carcinoma. However, this finding may not be evident, especially in small incisional biopsies, and CXPAs may often be misdiagnosed as PAs. Several studies have shown that the Ki-67 immunoexpression and other markers, such as HER2/neu, p53, androgen receptor, and BCL-2, are overexpressed in CXPAs compared with PAs (20,21). This data suggests that these molecules may play a role in the malignant transformation of PA and may serve as specific markers to distinguish CXPA from PA (20,21). In addition, fatty acid synthase and Ki-67 immunoexpression in combination have also been shown to be helpful for the identification of malignant components in CXPAs (22). Therefore, PAs must be carefully analyzed for atypical histopathological features, especially necrosis and prominent hyalinization, since studies have associated these findings with a risk of malignant transformation (15). In this study, an increase in mitotic activity, cellular pleomorphism, prominent hyalinization, and areas of necrosis was observed, reinforcing that such atypical characteristics are not expected in most PAs and should raise the suspicion of possible carcinomatous transformation. The suspicious cases were submitted to IHC for Ki-67, p53, and HER2/neu. They showed a high proliferative index and intense diffuse labeling for HER2/neu and p53 protein. These data reinforce the importance of careful morphological analysis and IHC in suspected cases to identify the carcinomatous component, ensuring a correct diagnosis.

Also, two AcNOS were reclassified as polymorphous adenocarcinomas. Of these, one was compatible with cribriform adenocarcinoma of minor salivary glands (CAMSG); and another was reclassified as a secretory carcinoma (SC). The latter was first described in 2010 as a mammary analog secretory carcinoma (MASC) and was recently recognized by the WHO (2). The SC represents a malignant tumor that was differentiated from AcCC and AcNOS because it shows significant similarity with the mammary-secreting carcinoma, besides presenting a specific translocation t(12;15)(p13;q25) that results in ETV6-NTRK3 gene fusion. According to the WHO, to standardize international nomenclature, the official designation for this entity is simply “secretory carcinoma.” In addition, although this tumor has an indolent clinical course like AcCC, it presents a higher probability of metastasizing to cervical lymph nodes (up to 25%) (2,23).

On the other hand, the CAMSG was first described by Michal *et al*. (1999) under the term cribriform adenocarcinoma of the tongue (CAT) (24). Years later, Skalova *et al*. renamed it “cribriform adenocarcinoma of minor salivary gland origin” because these tumors occurred in other oral sites such as the palate, retromolar region, tonsils, and upper lip (25). Currently, CAMSG is considered a possible variant of polymorphous adenocarcinomas due to morphological similarities (23). However, polymorphous adenocarcinomas have more diversified histology and nuclei with a characteristic “ground-glass” appearance (23,25). Also, although some CAMSG have shown an indolent clinical course similar to polymorphous adenocarcinoma, it presents a higher probability of metastasizing to cervical lymph nodes (1,23). Despite the regional aggressiveness of the CAMSG, differences in survival rates have not yet been established (1,23).

Molecular studies indicate that PRKD1-3 rearrangements, including ARID1A-PRKD1 and DDX3X-PRKD1 gene fusions, are seen in about 80% of CAMSG in contrast to polymorphous adenocarcinomas with classical morphology where less than 10% of cases show these changes (26). In comparison, PRKD1 E710D mutations are mainly seen in classical polymorphous adenocarcinomas, with only about 10% of CAMSG showing this mutation (27,28). The fact that genes of the same family drive both polymorphic adenocarcinoma and CAMSG suggests that they are variants of the same spectrum. For these reasons, the WHO decision was to maintain the CAMSG as a variant of polymorphic adenocarcinomas in the current version published in 2017 (2,23).

Despite all the changes proposed by the current edition of the WHO (2017) (2), it is essential to emphasize that the classification of SGTs is dynamic. With the recent advances in immunohistochemistry and molecular analysis, specific and refined changes continue to occur (1,29). Therefore, epidemiological studies are essential because they help improve the understanding of their clinical and pathological characteristics and keep physicians and surgeons updated when the classification of these tumors undergoes some change (1,3,4).

Regarding the anatomical location, the parotid gland was the most affected site (n = 82, 47.1%), followed by minor salivary glands of the palate (n = 45, 25.9%), like previous studies (3,5,9,12). However, some reports have shown that minor salivary glands of the palate are proportionally more affected by SGT than major salivary glands (4,6,19). In fact, in most studies derived from medical centers, the parotid gland is by far the most affected site, with 64% to 80% of all primary SGTs occurring at this site (3). Not surprisingly, studies conducted in oral pathology services have shown that intraoral minor salivary glands represent the most common site of these lesions (1). This difference maybe can be explained by the fact that most surgical specimens sent for oral pathology services are incisional biopsies or relatively small surgical specimens usually diagnosed and treated at primary and secondary services.

In contrast, most patients with SGT in the major salivary glands are often treated at hospitals and medical centers (1,6). Also, no benign or malignant tumor occurred in the sublingual gland in this study. The low prevalence and predominance of SGTs in the sublingual glands have been reported in the literature (1,3,8,12,13). However, when it occurs in this site, 70-90% of the tumors are malignant (2).

In conclusion, the data and results presented herein were similar to previously published reports in other countries and other world areas. However, in contrast to other studies, no striking age differences between malignant and benign SGTs were observed. Despite the rarity of these tumors, physicians and dentists must know the diversity of SGTs, thus contributing to the early diagnosis, effective treatment, and cancer prevention.
